# Prognostic and clinicopathological value of p53 expression in renal cell carcinoma: a meta-analysis

**DOI:** 10.18632/oncotarget.21971

**Published:** 2017-10-19

**Authors:** Zhun Wang, Shuanghe Peng, Ning Jiang, Aixiang Wang, Shuguang Liu, Hui Xie, Linpei Guo, Qiliang Cai, Yuanjie Niu

**Affiliations:** ^1^ Departments of Urology, Tianjin Institute of Urology, The second Hospital of Tianjin Medical University, Tianjin, 300211, China

**Keywords:** renal cell carcinoma, p53, prognosis, meta-analysis

## Abstract

**Background:**

The prognostic value of p53 expression in renal cell carcinoma (RCC) had been investigated in previous studies; however, the results remain inconsistent. This study was performed to investigate the prognostic and clinicopathological significance of p53 protein expression in RCC.

**Materials and Methods:**

Literature was identified from PubMed, Embase, Web of Science, and Cochrane database, which investigated the relationships between p53 expression and outcomes. Hazard ratios (HRs) for survival outcomes and odds ratios (ORs) for clinical parameters associated with p53 were extracted from eligible studies. Heterogeneity was assessed using the I2 value. The fixed-effects model was used if there was no evidence of heterogeneity; otherwise, the random-effects model was used. Publication bias was evaluated using Begg's funnel plots and Egger's regression test.

**Results:**

A total of 2,013 patients from 22 studies were included in the meta-analysis. The results showed that p53 positive expression is associated with poor overall survival (OS) (HR = 2.17, 95% confidence [CI]: 1.51–3.13) and cancer-specific survival (CSS) (HR = 1.59, 95% CI: 1.19–2.12) in RCC. In addition, p53 positive expression was closely correlated with TNM stage (III/IV vs. I/II: OR = 2.51, 95% CI: 1.05–6.00), Fuhrman grade (III/IV vs. I/II: OR = 1.80, 95% CI: 1.24–2.63), and distant metastasis (M1 vs. M0: OR = 1.70, 95% CI: 1.16–2.49), but not related to lymph node involvement (N1 vs. N0: OR = 1.32, 95% CI: 0.80–2.18), primary tumor stage (pT3/pT4 vs. pT1/pT2: OR = 1.16, 95% CI: 0.88–1.53), and sex (*n* = 2, male vs. female, OR = 1.09, 95% CI: 0.70–1.68).

**Conclusions:**

This study suggests that p53 positive expression is correlated with poor prognosis and advanced clinicopathological features in patients with RCC, which indicates that p53 is a potentially effective therapeutic target.

## INTRODUCTION

Renal cell carcinoma (RCC) is among the top 10 common cancers diagnosis in both men and women, which involve a heterogeneous group of cancers derived from renal tubular epithelial cells [1]. Approximately 295,000 new cases of RCC were diagnosed worldwide each year, with approximately 134,000 deaths [2, 3]. In the USA, there are approximately 63,000 new cases and approximately 14,000 deaths occur every year [4], with about 84,000 new cases and approximately 35,000 deaths in Europe [5]. There are several treatments available for local RCC, and the most effective method is surgery, followed by chemotherapy and radiotherapy. Nearly half of patients with RCC experienced disease recurrence after radical nephrectomy [6], and 30% of patients with RCC have metastases at the time of the initial diagnosis. Metastatic RCC (mRCC) is a treatment-resistant disease, usually treated with molecular-targeted agents or immune checkpoint blockade, but with limited efficacy [7]. Therefore, it is necessary to find a reliable prognostic biomarkers to distinguish high-risk patients with RCC, and improve the overall clinical outcome of patients.

p53, also known as tumor protein p53, cellular tumor antigen p53, or tumor suppressor p53, functions as a tumor suppressor [8]. p53 plays a role in apoptosis, genomic stability, and anti-angiogenesis. More than half of human tumors contain a mutation in the p53 gene, and p53 has become one of the most studied molecules in science [9, 10]. The degradation of the p53 protein is associated with binding of murine double minute 2 (MDM2). In a negative feedback loop, MDM2 itself is induced by the p53 protein, whereas the mutant p53 protein often fails to induce MDM2, resulting in the accumulation of p53 protein at very high levels. Moreover, the mutant p53 protein itself can inhibit normal p53 protein levels. In some cases, a single missense in p53 has been shown to disrupt the stability and function of p53 [11]. The p53 gene encodes a protein that binds to DNA, which in turn stimulates the expression of p21 protein and interacts with cell division-stimulating protein When p21 is complexed with cell division-stimulating protein, the cell stops the cell division process. Mutant p53 cannot be effectively bound to DNA , and the p21 protein cannot act as the “stop signal” for cell division, which results in tumors formation [12, 13]. It is reported that p53 expression predicts prognosis in various multiple cancer types including breast cancer [14], gastric cancer [15], multiple myeloma [16], colorectal cancer [17], cervical cancer [18], and oral cancer [19]. Many studies have investigated the prognostic role of p53 expression in RCC, but the results are conflicting [20–41]. Therefore, we conducted a comprehensive analysis to evaluate the prognostic and clinicalpathological value of p53 expression in patients with RCC.

## RESULTS

### Features of included studies

**Figure 1:**
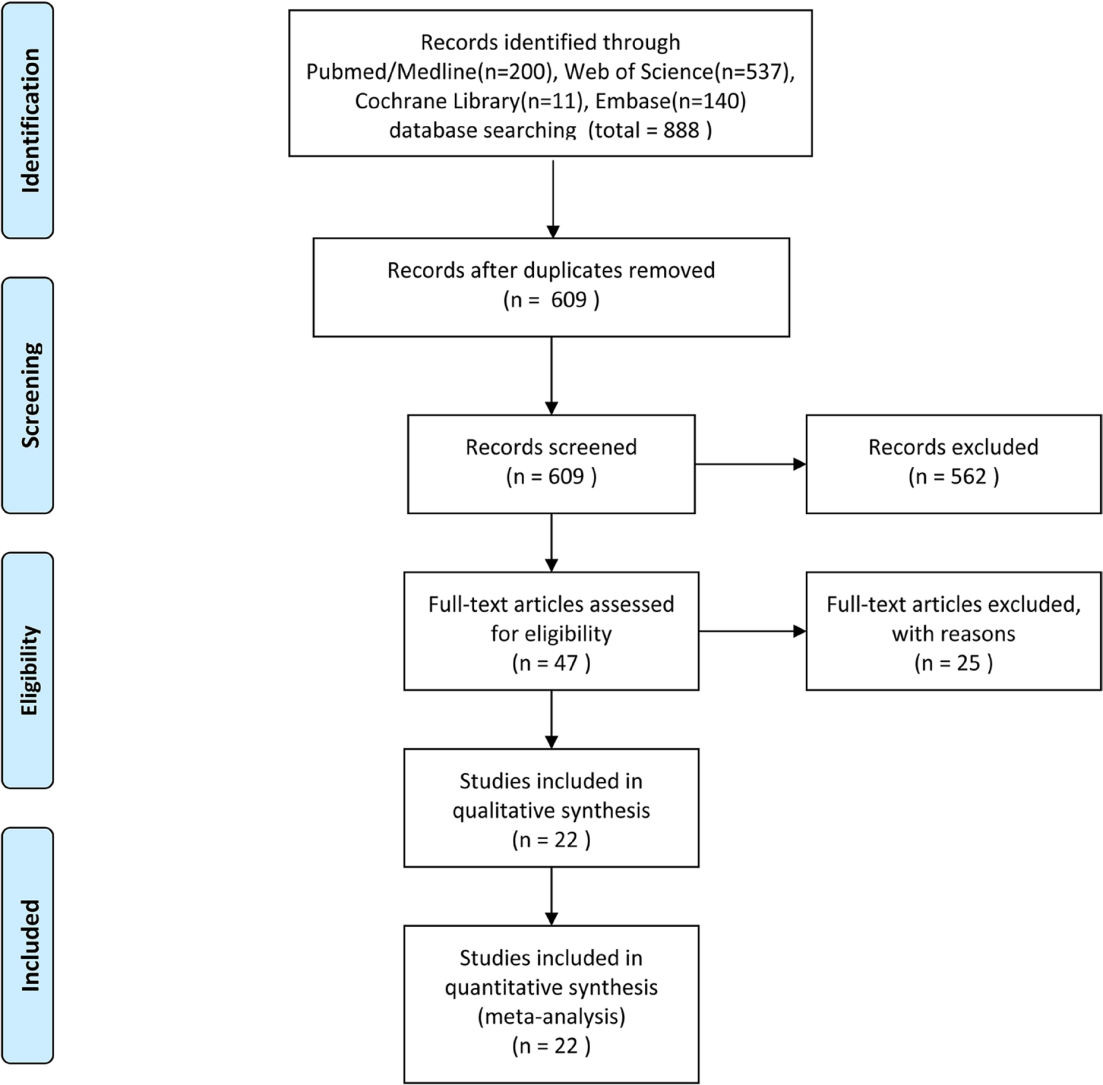
Flow diagram of the study selection process

A total of 888 potentially relevant studies were identified through systematic literature searches. After title and/or abstracts screening, 47 articles remained for full-text assessment. Then 25 articles were excluded (lacked key information). Finally, 22 studies [20–41] published from 1994 to 2015 with 2,013 patients that met our inclusion criteria were included in the meta-analysis (Figure 1). All studies were retrospective study design and detected p53 expression using IHC. The sample size ranged from 43 to 160. Eleven studies [20, 22, 25, 26, 29, 30, 32, 34–36, 38] were from western countries and eleven other studies [21, 23, 24, 27, 28, 31, 33, 37, 39–41] were from Asia countries. For the prognostic indicator of p53 expression in RCC, 2 articles reported both overall survival (OS) and cancer-specific survival (CSS), 13 articles reported OS, and seven articles reported CSS. Summary characteristics of these studies are shown in Supplementary Table 1.

### Prognostic value of p53 expression for OS and CSS

**Figure 2:**
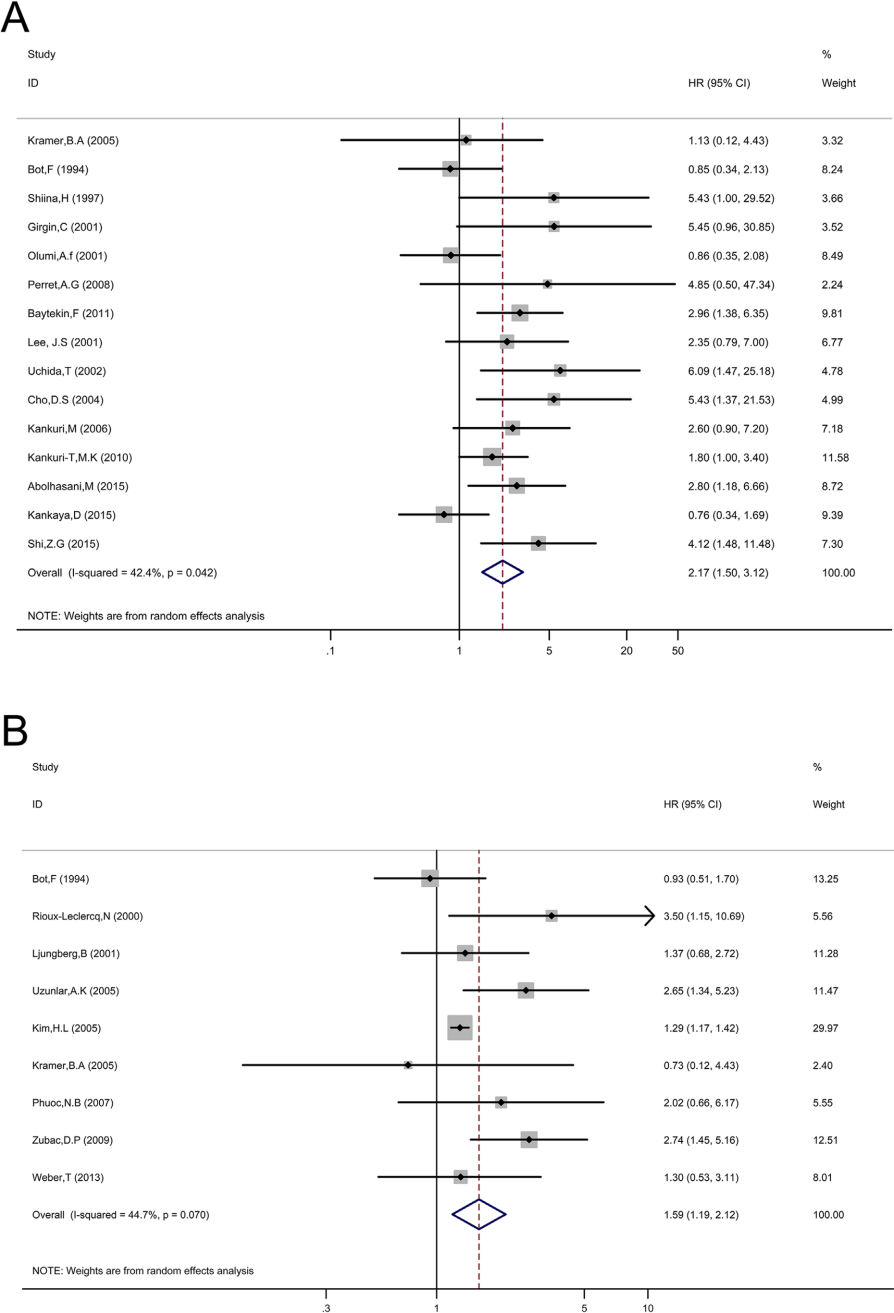
Forest plot HR for the correlation between p53 expression and OS (**A**) or CSS (**B**) in a patient with RCC.

The association between p53 expression and prognosis for OS and CSS in patients with RCC were estimated, the results of pooled hazard ratio (HR) and 95% confidence interval (CI) are shown in Table 1 and Figure 2.Fifteen studies evaluated the relationship between p53 expression and OS in patients with RCC. p53 positive expression was significantly associated with poor OS (HR = 2.17, 95% CI: 1.51–3.13, *p* < 0.001; I^2^ = 42.2%, P_heterogeneity_ = 0.042, Table 1, Figure 2). Subgroup analysis were performed according to HR estimate, nation and pathological types (Table 1). In subgroup analysis, the pooled HRs obtained from Kaplan–Meier curves (*n* = 6, HR = 2.04, 95% CI: 1.00–4.19, *p* = 0.052; I^2^ = 53.6%, P_heterogeneity_ = 0.056) and extracted directly from studies (*n* = 8, HR = 2.80, 95% CI: 1.18–6.66, *p* < 0.001; I^2^ = 44.6%, P_heterogeneity_ = 0.081) demonstrating that p53 positive expression was significantly associated with poor OS. With regard to nation, p53 positive expression was significantly correlated with poor OS (*n* = 5, HR = 4.08, 95% CI: 2.32–7.15, *p* < 0.001; I^2^ = 0.0%, P_heterogeneity_ = 0.042) in Asian patients compared with non-Asian patients (*n* = 10, HR = 1.68, 95% CI: 1.12–2.52, *p* = 0.012; I^2^ = 40%, P_heterogeneity_ = 0.091).

**Table 1:** p53 pooled HRs and 95%CIs in meta-analysis for OS and CSS

Stratified analysis	OS								CSS							
**No.of studies**	**Chi-squared**	**P_heterogeneity_**	**I^2^(%)**	**Pooled HR (95% CI)**				**No.of studies**	**Chi-squared**	**P_heterogeneity_**	**I^2^(%)**	**Pooled HR (95% CI)**			
				**Fixed effect**	***P*** **Value**	**Random effect**	***P*** **Value**					**Fixed effect**	***P*** **Value**	**Random effect**	***P*** **Value**
**Overall**																
	15	24.3	0.042	42.4	2.01 (1.55,2.61)	< 0.001	**2.17 (1.51,3.13)**	< 0.001	9	14.48	0.07	44.7	1.33 (1.21,1.46)	< 0.001	**1.59 (1.19,2.12)**	0.002
**Nation**																
Asia	5	1.56	0.042	0	**4.08 (2.32,7.15)**	< 0.001	4.08 (2.32,7.15)	< 0.001	1	-	-	-	2.02 (0.66,6.17)	0.218	2.02 (0.66,6.17)	0.218
Non-Asia	10	15	0.091	40	1.66 (1.23,2.22)	0.001	**1.68 (1.12,2.52)**	0.012	8	13.94	0.053	49.8	1.33 (1.21,1.454)	< 0.001	**1.57 (1.15,2.14)**	0.004
**HR estimate**																
Calculated	1	-	-	-	1.13 (0.19,6.84)	0.894	1.13 (0.19,6.84)	0.894	4	7.8	0.05	61.5	1.60 (1.06,2.40)	0.025	**1.70 (0.81,3.54)**	0.165
Directly	8	12.64	0.081	44.6	2.18 (1.57,3.03)	< 0.001	**2.80(1.18,6.66)**	< 0.001	4	5.3	0.151	43.4	1.31 (1.20,1.44)	< 0.001	**1.5 (1.072,2.101)**	0.018
Curves	6	10.77	0.056	53.6	1.79 (1.15,2.80)	0.01	**2.04 (1.0,4.19)**	0.052	1	-	-	-	2.02 (0.66,6.17)	0.218	2.02 (0.66,6.18)	0.218
**Histology**																
RCC	11	11.62	0.31	14	**2.36 (1.74,3.21)**	< 0.001	2.42(1.72,3.40)	< 0.001	4	5.62	0.132	46.6	1.40 (0.97,2.03)	0.074	**1.40 (0.81,2.40)**	0.227
ccRCC	3	7.35	0.025	72.8	1.21 (0.73,2.03)	0.46	**1.34(0.49,3,63)**	0.569	4	2.48	0.479	0	**2.27 (1.48,3.50)**	< 0.001	2.27 (1.48,3.50)	< 0.001
non-ccRCC	1	-	-	-	4.85 (0.50,47.19)	0.174	4.85(0.50,47.19)	0.174								
m-ccRCC									1	-	-	-	1.29(1.17,1.42)	< 0.001	1.59 (1.19,2.12)	< 0.001

OS: overall survival; CSS: cancer specific survival; HR: hazard ratios; 95%CI: 95% confidence interval.

Nine studies evaluated the relationship between p53 expression and CSS of patients with RCC. Pooled HR (1.59, 95% CI: 1.19–2.12, *p* = 0.002; I^2^ = 44.7%, P_heterogeneity_ = 0.007, Table 1, Figure 2) showed that p53 positive expression was also associated with poor CSS. p53 expression was also associated with poor CSS (HR = 1.59, 95% CI: 1.19–2.12, *p* = 0.002; I^2^ = 44.7%, P_heterogeneity_ = 0.007, Table 1, Figure 2). In subgroup analysis, the pooled HRs extracted directly from studies (*n* = 4, HR = 1.50, 95% CI: 1.07–2.10, *p* = 0.018; I^2^ = 43.4%, P_heterogeneity_ = 0.151) and calculated from demographic data (*n* = 4, HR = 1.70, 95% CI: 0.81–3.54, *p* = 0.165; I^2^ = 61.5%, P_heterogeneity_ = 0.05) demonstrating that p53 expression was significantly associated with poor CSS.

### Evaluation of p53 expression and clinicopathological characteristics

To explore the significance of p53 in pathologic diagnosis, we evaluated the correlation between p53 expression and clinicopathological features. The data of primary tumor stage, lymph node metastasis, distant metastasis, tumor node metastasis (TNM) stage, Fuhrman grade, and sex were extracted from the studies, and then the pooled OR and 95% CI were calculated.

**Figure 3:**
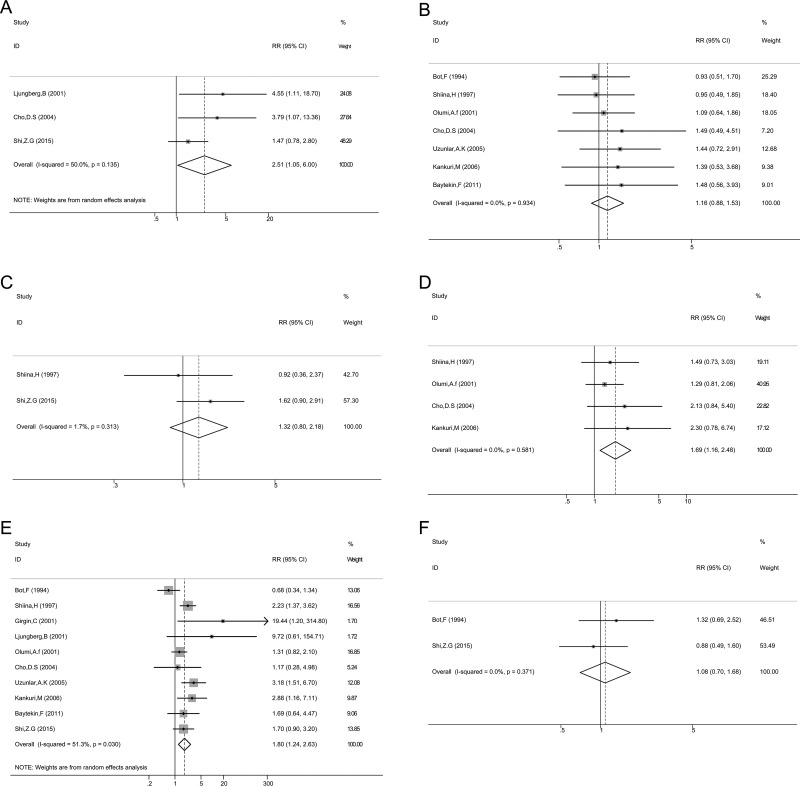
Association between p53 expression and TNM stage (**A**); primary tumor stage (**B**); lymph node metastasis (**C**); distant metastasis (**D**); Grade (**E**); Sex (**F**).

As shown in Figure 3 and Table 2. p53 expression was significantly associated with TNM stage (*n* = 3, III/IV vs. I/II, OR = 2.51, 95% CI: 1.05–6.00), Fuhrman grade (*n* = 11, 3/4 vs. 1/2, OR = 1.80, 95% CI: 1.24–2.63), and distant metastasis (*n* = 4, M1 vs. M0, OR = 1.70, 95% CI: 1.16–2.49). However, p53 positive expression was not associated with lymph node metastasis (*n* = 2, N1 vs. N0, OR = 1.32, 95% CI: 0.80–2.18), primary tumor stage (*n* = 7, pT3/4 vs. pT1/2, OR = 1.16, 95% CI: 0.88–1.53), and sex (*n* = 2, male vs. female, OR = 1.09, 95% CI: 0.70–1.68). The results indicated that p53 positive expression in patients with RCC could be considered as a biomarker to diagnose RCC in patients with higher grade, advanced stage, or distant metastasis.

**Table 2:** Meta analysis of p53 expression and clinicopathological features in renal cell carcinoma

	No.of studies	Chi-squared	P_heterogeneity_	I^2^(%)	Pooled OR (95% CI)				Begg’s Test *P* Value	Egger’s Test *P* Value
**Fixed model**	***P*** **Value**	**Random model**	***P*** **Value**
Tumor stage (pT3/pT4 vs pT1/pT2)	7	1.84	0.934	0	**1.16 (0.88,1.53)**	0.293	1.15 (0.87,1.50)	0.33	0.548	0.085
N (N1-2 vs N0)	2	1.02	0.313	1.7	**1.32 (0.80,2.18)**	0.275	1.38 (0.83,2.29)	0.209	1.000	-
M (M1 vs M0)	4	1.96	0.581	0	**1.70 (1.16,2.49)**	0.007	1.51 (1.08,2.13)	0.017	0.734	0.464
TNM (III/IV vs I/II)	3	4	0.135	50	2.62 (1.47,4.67)	0.001	**2.51 (1.05,6.00)**	0.039	0.296	0.263
Grade (3/4 vs 1/2)	11	18.46	0.03	51.3	1.84 (1.42,2.38)	< 0.001	**1.80 (1.24,2.63)**	0.002	0.386	0.175
Gender (Male vs Female)	2	0.8	0.371	0	**1.09 (0.70,1.68)**	0.717	1.06 (0.68,1.64)	0.798	1.000	-

Tumor stage:primary tumor stage; N:lymph node involvement; M:distant metastasis; TNM:TNM stage; 0R:odds ratio; 95%CI:confidence interval.

### Publication bias

**Figure 4:**
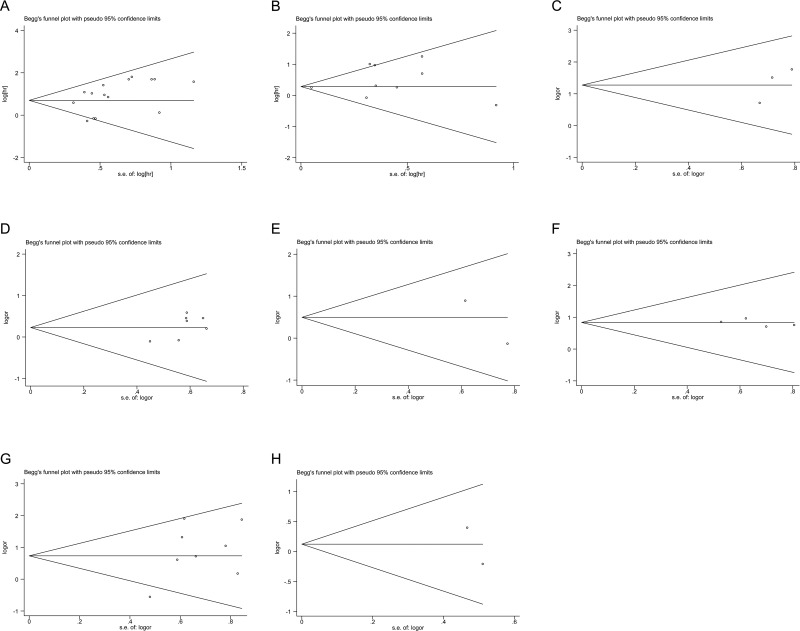
Funnel plots evaluating possible publication bias for OS (**A**); CSS (B); TNM stage (**C**); primary tumor stage (**D**); lymph node involvement (**E**); distant metastasis (**F**); grade (**G**); and sex (**H**).

Begg's funnel plots and Egger's test were used to assess the publication bias in this meta-analysis. Funnel plots for meta-analysis of p53 expression and OS and CSS are shown in Figure 4. Both the Begg's funnel plot test (OS: *p* = 0.235, CSS: *p* = 0.917; Figure 4) and the Egger's test (OS: *p* = 0.095, CSS: *p* = 0.203) verified the absence of any obvious publication bias. The funnel plots for clinical features also indicated no obvious publication bias (Table 2).

### Sensitivity analysis

**Figure 5:**
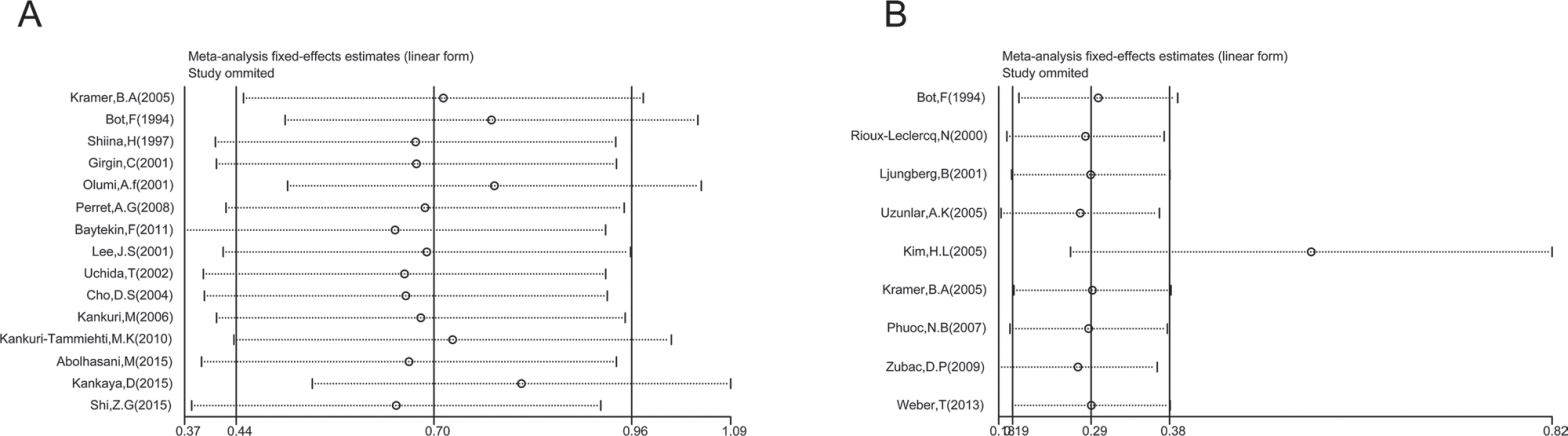
Sensitivity analysis in this meta-analysis (**A**) Sensitivity analysis for the p53 expression with OS. (**B**) Sensitivity analysis for the p53 expression with CSS.

**Figure 6:**
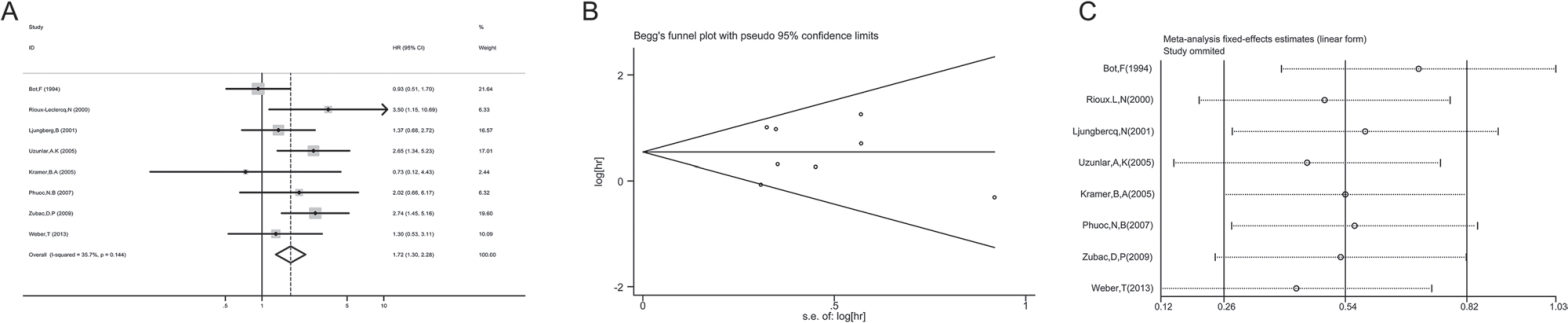
Meta-analysis of CSS following exclusion of data from Kim (2005) (**A**) Forest plot analysis of CSS. (**B**) Sensitivity analysis to confirmation of results’ stability. (**C**) Publication bias to the evaluation of studies’ symmetry.

A sensitivity analysis was performed to evaluate the stability of results, and to reduce the effect of the individual studies on final conclusions. The test suggested that for OS, the pooled result did not tend to exhibit alterations when an individual study was excluded (Figure 5). However, for CSS, the study by Kim (2005) had an obvious influence on the pooled result [29]. A more convincing pooled HR and 95% CI (HR = 1.72, 95% CI: 1.30–2.28, Figure 6A) was obtained when these data were excluded. The heterogeneity was decreased slightly (I^2^ = 35.7%, P_heterogeneity_ = 0.144). In addition, the results of sensitivity analysis and publication bias no longer changed (Figure 6b, 6C).

## DISCUSSION

p53 normally functions as a tumor suppressor [8], and p53 mutations exist in more than 50% of human tumors [9, 10]. Mutant p53 proteins usually causes the accumulation of p53 protein at a very high levels, and the mutant p53 protein itself can inhibit normal p53 protein levels [11]. In recent years, several reports have shown that p53 expression can serve as a promising biomarker for predicting various tumors [14–19]. Many studies have also reported the prognostic value of p53 expression in RCC, but the results were still conflicting [20–41]. Therefore, we performed this meta-analysis to explore the association between p53 expression and prognostic value in patients with RCC.

Our analysis mainly reports the prognostic role of p53 expression in RCC. Studies from different countries are included in the meta-analysis. Fixed-effect model and random-effect model were conducted according to the existence of heterogeneity. In this study, we focused on validating p53 immunohistochemical expression and evaluated the prognostic values of p53 expression in RCC. Based on results from 22 studies with 2,003 participants, we concluded that p53 positive expression predicted poor prognostic for patients with RCC. Those RCC patients with p53 positive expression exhibited poor OS and CSS. Subgroup analysis showed that the pooled HRs results obtained from Kaplan–Meier curves and extracted directly from studies demonstrated that p53 expression was significantly associated with poor OS and CSS. The relationship between p53 expression and clinicopathological features was also evaluated. The result suggested that patients with RCC and positive expression of p53 expression were significantly associated with nuclear grade, TNM stage, and distant metastases, but not with lymph node metastasis, primary tumor stage, and sex. To the best of our knowledge, this study is the first comprehensive analysis of the associations between p53 expression and prognostic significance in patients with RCC..

p53 plays a major role in human cancer by regulating the transcription of its downstream target genes [12, 13]. Mutations in p53 genes occur mainly in the DNA-binding domain, and dysfunctional p53 protein is accumulated in tumors [42]. Mutant p53 maybe a drug target for cancer therapy. Small molecule compounds which specifically target mutant p53 have been developed, including compounds that restore wildtype p53 transcriptional activity and reduce mutant p53 levels, which indicate that target mutant p53 may be an effective strategy for cancer therapy [43].

Our results suggested that p53 positive expression was an unfavorable predictor for prognosis in RCC, which was in accordance with conclusions determined from other solid cancer types, such as breast [14], gastric [15], colorectal [17], cervical [18], and oral [19].

We also analyzed the association between p53 expression and clinical factors in RCC, and the results suggested that p53 positive expression was closely related to higher tumor stage and grade, as well as distant metastases, which indicated that p53 had potential to be a dichotomous biomarker.

mRCC is a treatment-resistant malignant tumor, though targeted agents and immune checkpoint blockade have been used for mRCC, but with limited efficacy [7]. The therapeutic strategy of mRCC with p53 positive expression may target mutant p53 to improve clinical outcomes.

There are several limitations should be acknowledged. First, all included studies in this meta-analysis measured p53 expression by immunohistochemistry, but the cut-off criteria to determine the positive or negative expression of p53 and the primary antibodies used for detected p53 expression were inconsistent in different studies, which may potentially contribute to heterogeneity. Therefore, a more unified standard should be defined in the future. Second, the number of patients included in the most eligible studies was relatively small. Therefore, large scale studies are needed to conceive more reliable results. Third, relatively few studies were extracted in some subgroup analyses, which might render premature results. Finally, research with positive results is potentially more likely to be submitted and published than work with negative results, which could cause publication bias, although this bias was not detected in the present analysis [44].

In conclusion, our meta-analysis suggests that p53 expression predicted a poor OS and CSS in patients with RCC. The results also indicate p53 expression was associated with more aggressive clinical features in patients with RCC. More prospective and large scale studies are needed to clarify our results.

## MATERIALS AND METHODS

### Search strategy and selection criteria

We did this meta-analysis using a predefined protocol in accordance with PRISMA [45]. We searched PubMed, Embase, Web of Science, and the Cochrane electronic databases for studies published before April 14, 2017. The computer-based searches combined terms related to “renal cell carcinoma” or “renal cell cancer” or “renal cell adenocarcinoma” or “kidney tumor” and “p53” and “prognosis” or “survival” or “outcome” in humans; the language of publications was restricted to English.

Two reviewers (WZ and LSG) independently screened the titles and abstracts of all initially identified studies according to the selection criteria. Full-text articles of studies that met all selection criteria were retrieved.

The eligible studies must meet the follow criteria: (1) all patients received a diagnosis of histologically confirmed RCC; (2) the prognostic value of p53 expression for OS and/or CSS were reported; (3) HRs and their 95% CIs for survival analysis were reported in the text or could be computed from given data; (4) the expression of p53 was measured by immunohistochemistry; The exclusion criteria were as follows: abstract, review, case report or comment letter; animal studies; duplicate publications; published not in English.

### Data extraction and quality

Two authors (WZ and PSH) independently extracted data and a consensus was reached in case of any inconsistency with the involvement of a third author (CQL). We used a predesigned data extraction form to obtain relevant information. The data extracted from the eligible studies, included the following items: first author, year of publication, country of origin, number of patients, histopathological stage, detection method, cut-off value, antibody for p53 staining, number of positive p53 expression, HR for survival (OS and/or CSS), and follow-up time. For articles that only provided survival data in a Kaplan–Meier curve, software designed by Jayne F Tierney and Matthew R Sydes was used to digitize and extract the OR and its 95% CI [46].

### Statistical analysis

Data were analyzed using Stata SE12.0 (Stata Corp LP, College Station, TX, USA). The associations between clinical factors and p53 expression were presented by OR and 95% CI. HR with a 95% CI was computed to reveal the correlation between p53 expression and prognosis (OS and CSS). Interstudy heterogeneity was evaluated using the chi-square test and I^2^ statistic (100% x [(Q-df)/Q]) [47, 48], the value of P_heterogeneity_ < 0.1 and I^2^ > 50% represents significant heterogeneity, and the value of P_heterogeneity_ > 0.05 and I^2^ < 25% represents low heterogeneity. A fixed effects model was used when the value of P_heterogeneity_ > 0.05 and I^2^ < 25%; otherwise, a random-effects model was applied. Subgroup analysis was performed for OS and CSS analysis. Begg funnel plot and Egger linear regression tests evaluated the potential for publication bias. Two-tailed value of *p* < 0.05 was considered statistically significant.

## SUPPLEMENTARY MATERIALS TABLE




