# Correction: Prognostic and clinicopathological value of p53 expression in renal cell carcinoma: a meta-analysis

**DOI:** 10.18632/oncotarget.26394

**Published:** 2018-11-20

**Authors:** Zhun Wang, Shuanghe Peng, Ning Jiang, Aixiang Wang, Shuguang Liu, Hui Xie, Linpei Guo, Qiliang Cai, Yuanjie Niu

**Affiliations:** ^1^ Departments of Urology, Tianjin Institute of Urology, The second Hospital of Tianjin Medical University, Tianjin, 300211, China

**This article has been corrected:** The correct Funding information is given below:

**FUNDING**

This meta-analysis has been financially supported by the National Natural Science Foundation of China (No. 81472682 and No. 81572538) and the Science Foundation of Tianjin (No. 16JCZDJC34400).

Original article: Oncotarget. 2017; 8:102361-102370. https://doi.org/10.18632/oncotarget.21971

