# Supplementary material for: Prognostic and clinicopathological value of p53 expression in renal cell carcinoma: a meta-analysis
Source: Oncotarget. 2017 Oct 19;8(60):102361–70. doi: 10.18632/oncotarget.21971 (PMC5731961; doi:10.18632/oncotarget.21971)
Supplement: Supplementary file 1 [file oncotarget-08-102361-s001.pdf]

Prognostic and clinicopathological value of p53 expression in renal cell carcinoma: a meta-analysis

SUPPLEMENTARY MATERIALS

Supplementary Table 1: Main characteristics of included studies. See_Supplementary_Table 1
